# A polyp-on-chip for coral long-term culture

**DOI:** 10.1038/s41598-020-63829-4

**Published:** 2020-04-24

**Authors:** Ai-Ping Pang, Yongsheng Luo, Chunpeng He, Zuhong Lu, Xiaolin Lu

**Affiliations:** State Key Laboratory of Bioelectronics, School of Biological Science & Medical Engineering, Southeast University, Nanjing, 210096 Jiangsu Province P.R. China

**Keywords:** Lab-on-a-chip, Biomedical engineering

## Abstract

Coral polyps are basic clonal biological units of reef corals. However, *in vitro* experimental model for long-term physiological and ecological studies has not been well developed due to the difficulty of effectively acquiring and culturing single polyps. This study developed an experimental platform based on microfluidics for culturing single coral polyps and tracing its growth state over time in the long run. The corresponding computational modeling was conducted to predict the metabolic processes under the static and dynamic conditions by coupling the mass transfer and reaction with Navier-Stokes equations. Design and fabrication of the microfluidic chip was the key to provide a constant laminar flow environment that enabled the controlled high oxygen and bicarbonate transfer for the cultivation of the single coral polyps. The single coral polyps were induced to bail out of the coral reef upon the chemical stress and cultured for more than fifteen days in the microfluidic chip. It was found that the single coral polyps in the microfluidic chip can maintain their normal metabolic process over the cultivation period, suggesting that our microfluidic platform can serve as a suitable tool to study the coral polyps by providing a controllable and suitable biological microenvironment.

## Introduction

Stony corals are essential building blocks for coral reefs in marine ecosystems by providing foods, habitats and shelters for a large number of marine organisms. In recent years, coral bleaching occurred more and more frequently due to the environmental deterioration, for example ocean acidification and greenhouse effect^1,2^. It is thus urgent to conduct studies related to both the physiological and ecological processes of reef corals for the purpose of maintaining the biodiversity, protecting and restoring the marine ecosystems, and defending the sensitive shorelines from the waves^1,3,4^. To date, a series of investigations have been carried out on the physiology, symbiosis (with zooxanthellae) and biomineralization of the reef corals at the cell and tissue levels^5–10^. Although experimental methods for primary cultures have been established, the subsequent efficient proliferation of such cnidarian cells is still a problem. This is more or less due to lack of the experimental approaches to screen the key factors which affect the metabolic process of reef corals in a microscopic level.

To this end, it has been suggested that studying the coral polyps, which serves as model organisms for reef corals, is a more plausible way to acquire the necessary physiological information to understand the biological behaviors of reef corals^11–15^. However, it still remains a challenge to collect and culture single coral polyps *in vitro*^16^. Usually there are two approaches to obtain single coral polyps. One is to collect the planulae larvae and induce its metamorphosis to a primary polyp^12,17^. Normally a few nights around the full moon in an appropriate season are needed. This method is time consuming and restricted by the coral spawning period due to individual differences. The other one is to induce the detachment of the single polyps from the coral skeletons. For both methods, the main problem is how to create a suitable *in vitro* environment to support the long-term survival of the coral polyps. Although it has been reported that the polyps of solitary coral *F. granulosa* can survive in the glass Petri dishes for over three years^18^, the most majority of stony corals are colonial ones, which are different from solitary corals in the polyp size, structural and cultural features. And, as mentioned above, the single coral polyps bailing out of the coral skeletons are so sensitive to the environment that it is difficult for them to be cultured *in vitro* for a long period of time^19^. Previous studies indicated that culture of the single coral polyps suspended in the solution was limited to a week with two days’ seawater renewal^16^.

In order to solve the above problem, a biomimetic microwell-based microfluidic chip platform is presented here for the long-term culture of the single coral polyps, which can conveniently be combined with the microscopy. Compared with the traditional culture method, the microfluidic culture platform is advantageous in feasibly and precisely controlling the local cellular microenvironment and monitoring the growth state of the cultured living organisms in real time and *in situ*^20–22^. The platform can provide an adjustable microenvironment to control the temperature, light intensity and fluid composition, which can easily be assembled and disassembled based on different experimental requirements. To ensure the feasibility and robustness, the flow characteristics and the mass transfer for the dissolved oxygen (DO) and dissolved inorganic carbon (DIC) in this chip were evaluated and analyzed by the computer simulation. Single coral polyps were bailed out and cultured in this dynamic microfluidic chip under the traditional static and the flowing conditions. Experimental results confirmed that this coral-polyp-on-chip methodology is a more efficient approach to support the *in vitro* long-term culture of single coral polyps.

## Materials and Methods

### Coral microchip fabrication

**Figure 1:**
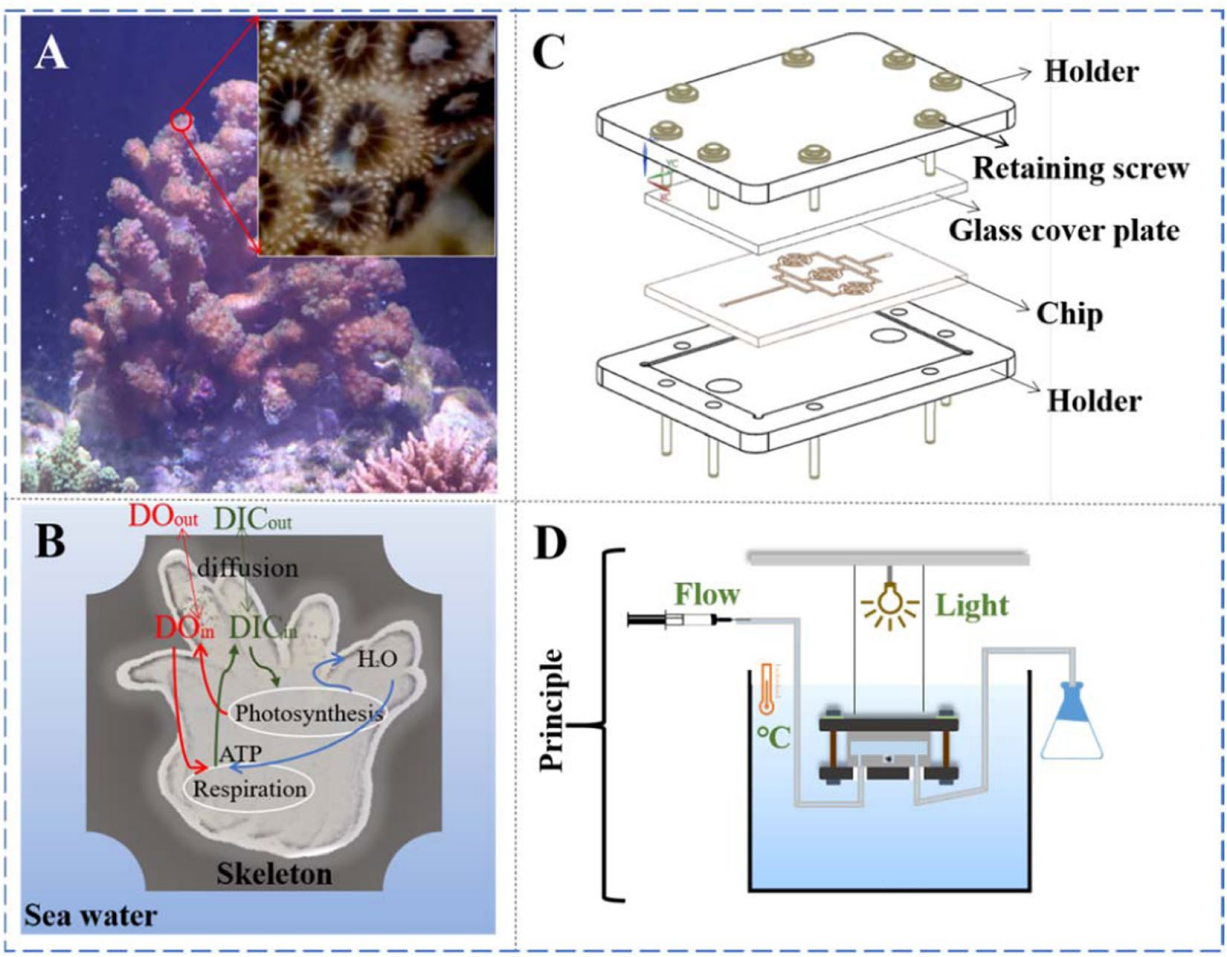
Schematic shows the concept of this coral-polyp-on-chip for the culture of single coral polyps. (**A**) An *in vivo* coral reef in shallow water which serves as a basis to extract necessary parameters for designing a biomimetic chip. (**B**) The metabolism model of a coral polyp, mainly involved in the transport and diffusion of DO and DIC. (**C**) Layout of designed 3D microchip. (**D**) Working principle for *in vitro* culture of single coral polyps using the coral-polyp-on-chip immersing in the simulated seawater environment.

Several aspects need to be taken care of when the microchip system was designed. First, it should be transparent in terms of the visible light in order for the coral polyps to be observed and imaged. Second, a dynamic fluid microenvironment should be considered to provide enough nutrients and remove unnecessary metabolic wastes. Third, the temperature and light intensity need to be changed within the chip as adjustable parameters. Therefore, as shown in Fig. 1C, a chip with a multilayer structure was employed, including a polymethyl methacrylate (PMMA) substrate, a cover glass, and a holder. The PMMA substrate contained three micro-cavities in parallel connected by flow channels with the fluid going in and out using silicone tubes. A syringe pump was connected to the chip to keep the fluid flowing at a constant speed. The cover glass was mounted onto the substrate after the explantation of the coral polyp. The two PMMA base holders (overall thickness ~ 4 mm), fixed by eight screws at the edge area, were used to seal the cover and substrate, thus allowing this multilayer microchip to be assembled and disassembled feasibly.

The multilayer chips were fabricated via the micromachining technology. In brief, the template was designed using the Software AutoCAD 2014 and processed by a computer controlled milling machine. On the PMMA substrate (thickness ~2 mm), the channels were engraved with a width of 1 mm and a height of 300 μm. The microwell was created on the substrate with a height of 500 μm and a diameter of 1.5 mm. Before the experiment, all the layers were sequentially rinsed with ethanol (Kermel), ultrapure water (18.2 ${\rm{M}}\Omega$, Milli-Q gradient A10 water purification system, Millipore Corporation), and finally blew dry with N_2_.

### Modeling of flow characteristics, DO and DIC mass transfer in the chip

Photosynthesis and respiration are two critical biological processes in the long-term culture of coral polyps, which are associated with the uptake and release of the DO and DIC^23–26^. The DIC in the coral coelenteron is mainly taken in by symbiotic zooxanthellae for the photosynthesis to generate the photosynthates (DO, ${{\rm{H}}}_{2}{\rm{O}}$) upon consuming equal molar numbers of DIC (Fig. 1B). The photosynthates are then used for respiration with the product (DIC) stored in coelenteron. However, coral polyps still need extra DIC from the ambient seawater^27^ since the photosynthesis rate of zooxanthellae algae is higher than that of coral polyps. To know the details about the flow environment in the chip, the flow characteristics, the DO and DIC transfer were simulated using the COMSOL Multiphysics 5.2a software. The simulated area included a 300 μm deep, 2 mm wide channel with six circle columns and a 500 μm deep microwell (radius 750 μm), where a single coral polyp settled. First, the flow field distribution of the liquid flow in the channel was evaluated to identify the key design parameters. The DO and DIC transfer and consumption were analyzed based on the flow field. Simulation details were put in the Supporting information.

### Bail-out and explantation of single coral polyps

Based on the calcium-free cell dissociation protocol^5^, the single coral polyps of *P. damicornis* (Coelenterata, Anthozoa, Scleractinia, collected from the coral reef in Wenchang, Hainan Province, China) were successfully induced to bail out of the coral reef. Briefly, fast-growing apical fragments (length ~ 1.0 cm) of the coral reefs were excised from parent coral colonies and cut into the smaller ones (length ~ 0.3–0.5 cm) with a stainless tweezer. The fragments were then submerged in the calcium-free artificial seawater (CaFSW) in a Petri dish, washed three times and pre-incubated in CaFSW with an orbital incubator (80 rpm) for 3 h. Afterwards, the fragments were transferred to a culture medium (5 mL) supplemented with the 3% penicillin-streptomycin solution in a 6-well plate. Culture mediums with different Dulbecco’s modified Eagle’s medium (DMEM) contents were tested to screen the best condition for the coral polyp explantation. The plate was placed in a 26 °C incubator and monitored continuously for the bail-out of the single coral polyps. The primary cultures were changed every day until the single coral polyps were released from the fragmental skeleton. The bailed-out coral polyps were examined under a stereomicroscope for their integrity. The intact coral polyps, including a mouth, twelve tentacles and mesenteries were submerged in the filter-sterilized artificial seawater (FASW) for the initial recovery. Viable coral polyps were collected by a 1 mL pipette with carefulness and placed in the chip microwell at a flow rate of 90 ${\rm{L}}\,{{\rm{\min }}}^{-1}$, as shown in Fig. 1D.

### Culture of single coral polyps in the microfluidic chip

The microfluidic chip system was placed in a 525-liter glass aquarium filled with the artificial seawater. The culture process went with the temperature of 26 °C, salinity of 35 p.p.t (g L^−1^), 12/12 h illumination/dark cycle (white/red/blue fluorescent bulbs), and pH of 8.0–8.2. The *P. damicornis* colonies were acclimated for at least two weeks before experiments.

The artificial seawater was prepared by dissolving the coral pro salt (Red Sea, Europe) in a certain volume of deionized water until the salinity reached 35 p.p.t. The CaFSW was prepared by adding 23 g of NaCl, 0.763 g of KCl, 1.89 g of MgSO_4_·7H_2_O, 10.45 g of MgCl_2_·6H_2_O, 3 g of Na_2_SO_4_ and 0.25 g of NaHCO_3_ to a liter of deionized H_2_O. The pH value was adjusted to be 8.0 using the 1 M NaOH solution. The stock solution was prepared by dissolving 10 g of powered DMEM (GIBCO) in a liter of CaFSW, supplemented with 3.7 g of NaHCO_3_. A series of culture mediums were prepared in filter-sterilized artificial seawater (FASW) with different concentrations of stock solutions (0%, 20%, 40%, 60%, 80% and 100%). The penicillin-streptomycin solution (HyClone) was added when necessary.

### Characterization of coral polyps

Autofluorescences resulted from the native green fluorescent protein (green) in the *P. damicornis* coral polyps and algal chlorophyll (red) in the zooxanthellae^11^ can facilitate the fluorescence imaging. In this study, a laser scanning confocal microscope (LSCM, Leica TCS SP8, Germany) was used to image the coral polyps with the built-in software (Leica Application Suite X). The gross morphologies and fine structures of the coral polyps were characterized with the stereomicroscope (Olympus SZ51, Japan) and inversion fluorescence microscope (Olympus IX81, Japan). To capture the scanning electron microscopic (SEM, Scanning electron microscope, Hitachi SU8010) images, individual coral polyps were solidified and fixed in 2.5% glutaraldehyde for 1 h. The fixed coral polyps were then dehydrated upon immersion in pure ethanol, dried under vacuum, sputter-coated with gold-palladium and finally imaged.

## Results

### Bail-out and microscopic characterization of coral polyps

The coral polyp expulsion was often associated with the environmental stress. A number of stimuli have been adopted to induce the bail-out of the coral polyps, such as low pH, salinity gradient, thermal stress, chlordecone stress, or seawater with low nutrient load^11,28–31^. In this study, a new approach was employed.

**Figure 2:**
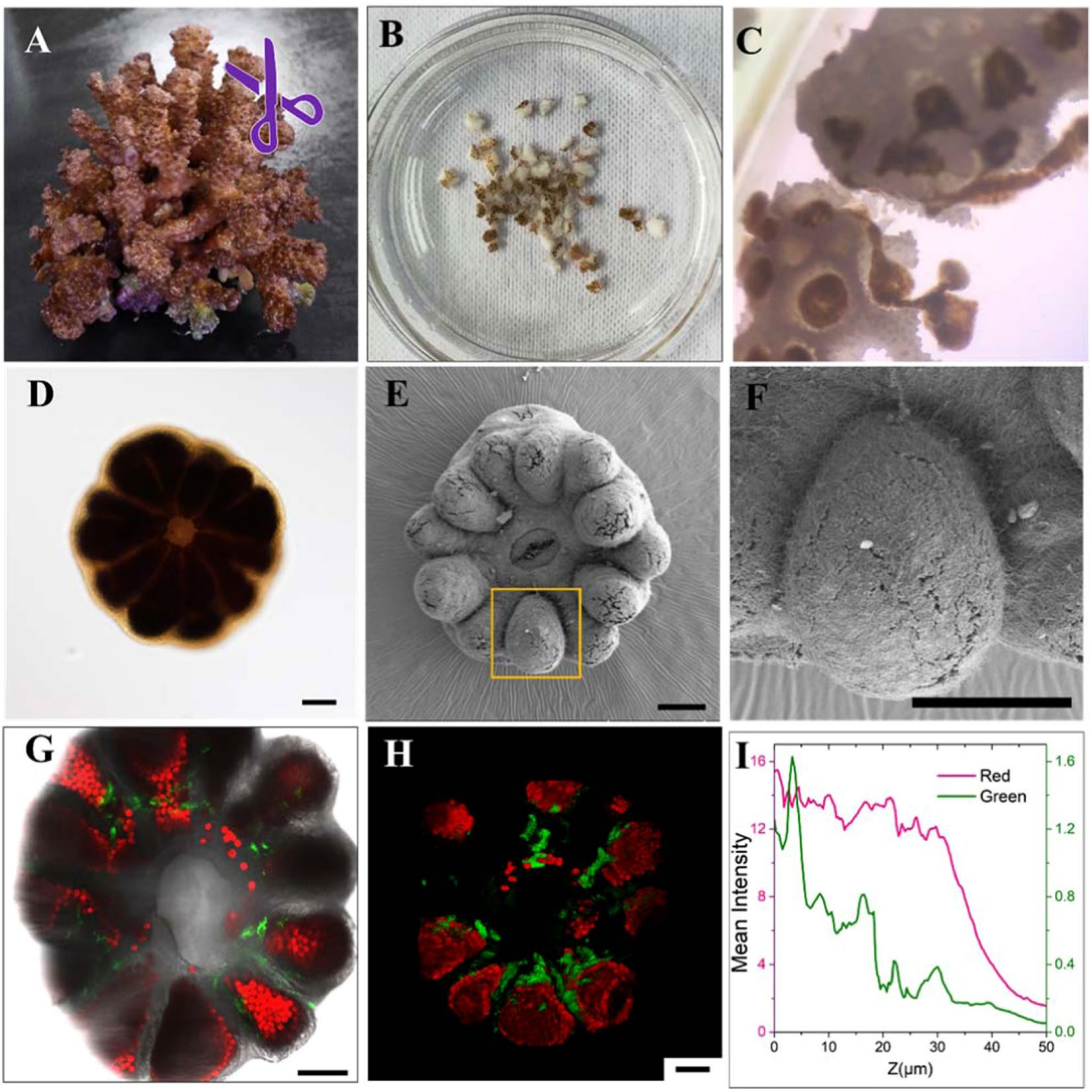
Bail-out of *P. damicornis* coral polyps and fluorescence imaging characterization. (**A**) Apical fragments (~1.0 cm) were excised from fast-growing parent coral colonies (**B**) and cut into small branches (0.3-0.5 cm) with stainless tweezers. (**C**) Detachment of the coral polyps from the parent coral skeleton. (**D**) A coral polyp under the Olympus IX81 microscope. A radial symmetry with twelve tentacles was clearly observed. (**E**) SEM image of an intact coral polyp. (**F**) High magnification view of the area marked with the orange rectangle in (**E**) shows a polyp’s tentacle. (**G**) A fluorescence image due to the autofluorescences from the coral native GFP (green) and algal chlorophyll (red) with LSCM. (**H**) The confocal microscopy-based 3D reconstruction of a coral polyp. (**I**) Mean fluorescence intensities for GFP (green) and algal chlorophyll (red) in terms of the z direction. The start position of the horizontal axis was set to the top layer from the oral end. Scale bars: 100 ${\rm{\mu }}{\rm{m}}$.

The expulsion of the coral polyps was induced from *P. damicornis* fragments (Fig. 2A). Six different culture mediums with the DMEM contents ranging from 0 to 100% were used. The process involved the withdrawal of individual coral polyps from the coenosarc followed by the detachment and release from the fragmental skeleton. We found, the withdrawal and detachment of the coral polyps were dependent on the DMEM concentration and the best bailed-out concentration was 20%. At this concentration, the individual coral polyps showed the complete biological structure and the high vitality under a stereomicroscope (See the Supporting Information, Fig. S1). Although culture mediums of higher DMEM concentrations (40%, 60%, 80% and 100%) led to the detachment of the coral polyps from their parent coral skeletons over shorter period of time, the surface epitheliums of the ectodermal layers for the released coral polyps were incomplete or missing (Fig. S1, taking 80% as an example). Given even longer time, pieces of tissues composed of aggregated coral polyps with the interconnected coenosarcs were released from the skeletons, again suggesting that the high concentrations were inappropriate for the explantation of the single coral polyps. This indicated that the moderately concentrated medium (20%) was already enough for the coral polyp expulsion, which allowed for both the active polyp detachment and tissue structural integrity at the same time. Additional micro-scale features for the oral and aboral ends of the coral polyps were observed under the SEM (Figs. 2E,F; S2 A–C). Around the mouth area, there existed several different kinds of algae. A previous study indicated *P. damicornis* could consume and digest algae to some extent^32^. The fluorescence images were captured for the cultured *P. damicornis* polyps under the LSCM (Figs. 2G,H; S2 D–F). As mentioned above, the autofluorescences from both the native green fluorescent proteins (GFP) in the coral polyp and algal chlorophyll made the imaging process feasible. As shown in Fig. 2G, the fluorescent images confirmed that zooxanthellae symbionts only existed in the coral polyp’s ectoderm. It should also be noted, this was the first time for the individual coral polyp’s 3D structure to be imaged using the GFP and chlorophyll fluorescence signals at a high spatiotemporal resolution (Fig. 2H) (Movie S1). Using the built-in software, the curves of the fluorescence intensities (red and green) as a function of z (depth) were plotted (Fig. 2I). The fluorescence intensity of algal chlorophyll (red) were very high for the first 0–300–30 ${\rm{\mu }}{\rm{m}}$ and went through a sharp decrease afterwards. This should come from the fact that zooxanthellae algae were located at the surface area of the mouth, where it was easy for them to acquire illuminance for the photosynthesis, thus providing more energy and oxygen for the growth of the coral polyp.

### Designing a microfluidic platform

**Figure 3:**
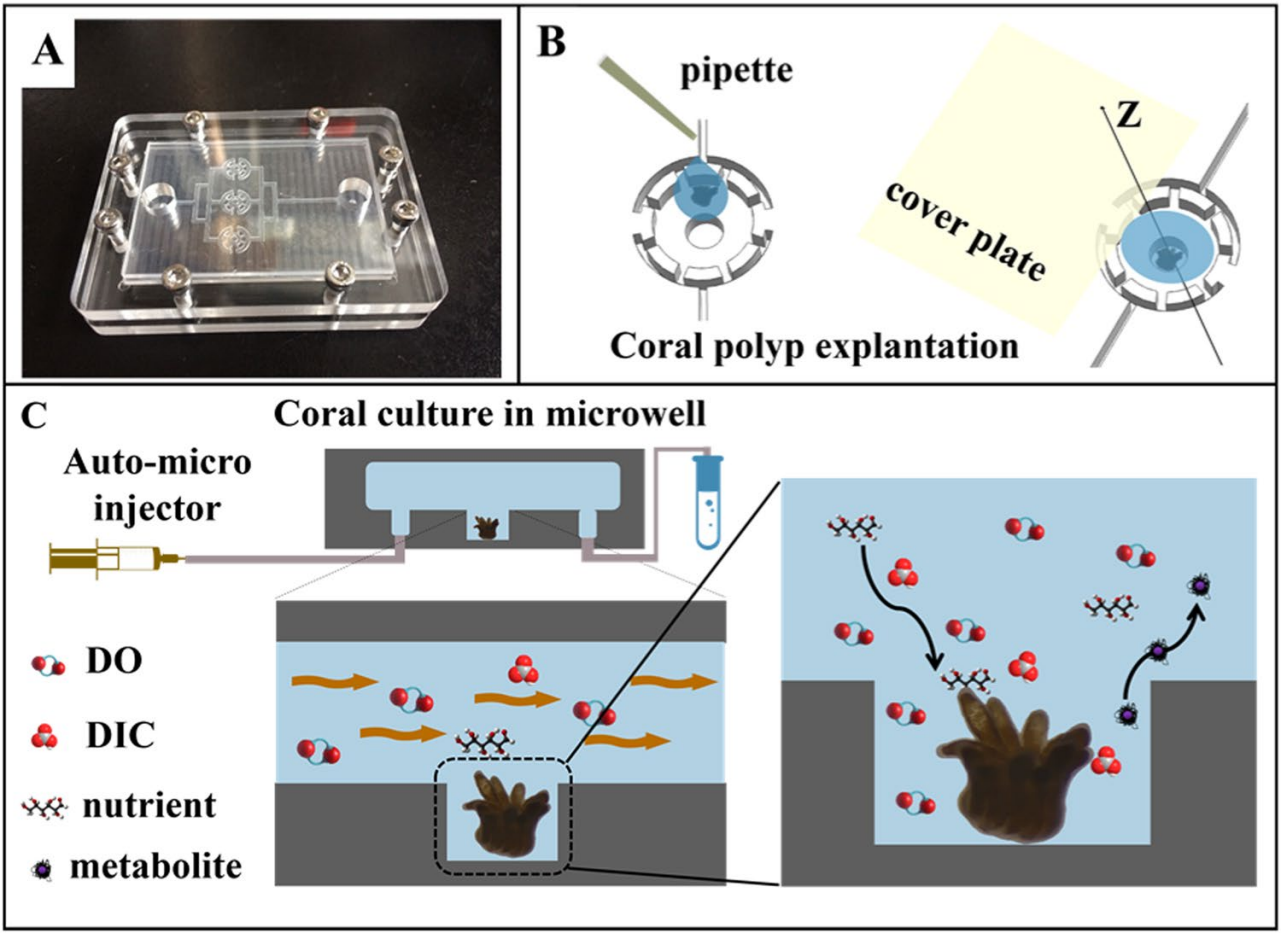
Culture of single coral polyps in the microchip. (**A**) Photo of the microfluidic chip with three microwells. The microchip was sealed with the cover glass using eight screws at the peripheral area. (**B**) Schematic presentation of the explantation of the coral polyp in the microwell. (**C**) A micro-pump was used to provide a fluid environment for the coral polyp culture. Design of the microwells in the chip can allow for a rapid exchange of soluble substrates (DO, DIC, nutrients, and waste) and to reduce the effect of shear stress on the single coral polyps.

As the core component of the coral polyp culture, the assembled microfluidic chip platform consists of three micro-cavities (or microwells) used to settle single coral polyps with the fluid flowing in the channels (Fig. 3A). The single coral polyps could easily be transferred into the chip by pipette transplanting, as shown in Fig. 3B. The problem of generating air bubbles could be avoided by filling the channels with the fluid before transplanting the coral polyps. In this way, the coral polyps could be settled in the microwells without injury. Owing to the disassembility of the microchip system, the cultured coral polyps can also be taken out of the microchip for further characterization, for example SEM. To maintain the normal metabolism of the coral polyps, the environmental factors including the flow rate, temperature and light intensity were precisely controlled in the aquarium.

A vital factor to be considered for the chip design is the structure and size of the microwells. If the microwell is too shallow, the coral polyps may bear the excessive impact from the liquid flow, which adversely affects the growth of the coral polyp. On the contrary, if the microwell is too deep, the effective mass transfer for the sufficient nutrient supply for the coral polyps cannot be guaranteed. Therefore, the depth and diameter of the microwell were selected to be 500 ${\rm{\mu }}{\rm{m}}$ and 1.5 mm based on our simulation result. At the peripheric area of the microwell, there was a circular channel with 200 ${\rm{\mu }}{\rm{m}}$ in depth and 3 mm in diameter to buffer the fluid impact on the coral polyp, thus preventing the coral polyp from entering the top channel (see the Fig. S3 for detail).

### Modeling of metabolic process

**Figure 4:**
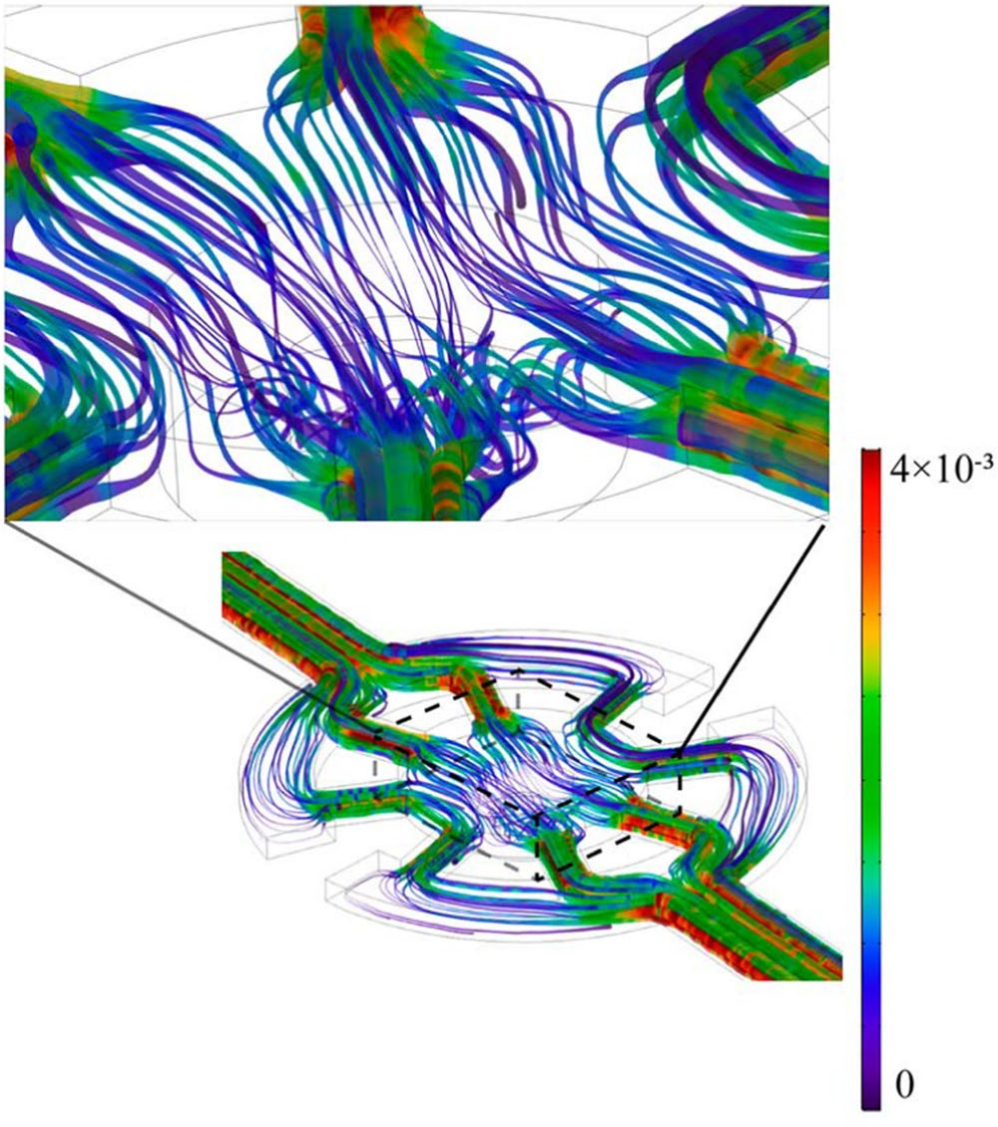
The flow field in the microwell. Distribution of velocity and shear rate obtained from the numerical simulation are described as a 3D representation. The flow velocities are represented by the different colors and the shear rates are represented by the thicknesses of the flow lines.

The flow characteristics in the microfluidic chip were crucial for the coral polyp’s long-term survival and growth. Two important parameters must be considered, i.e. the flow velocity and the shear rate, which are directly associated with the mass transfer and the impact force regarding the coral polyp. In order to balance the mass transfer and the impact force, the combined effect of both convection and diffusion need to be considered. Generally speaking, if the Peckert number is larger than 1, the convection contributes to the mass transfer more than the diffusion does. The homogeneous mass distribution can effectively be achieved by continuous perfusion. From the simulation results of various inlet rates (15–120 ${\rm{\mu }}{\rm{L}}/\,{\rm{\min }}$) (Supporting Information, Fig. S3), the streamline plot of the velocity distribution in the microwell at the optimum inlet flow rate, $Q=30\,{\rm{\mu }}{\rm{L}}\,{{\rm{\min }}}^{-1}$, was shown in Fig. 4. As the liquid flowed forward through the system, the low velocity streamlines were distributed (blue) at the center area of the microwell and near the grooves, thus presenting the lower shear rates than those in the channels (red and green). The shear rate of the coral reef in the natural environment is in a range of 0.11–3 s^−1^ ^33,34^. Our simulation result has shown that the shear rate in the chip environment (1.5–2.5 s^−1^) is within this range. The simulation results clearly indicated that the velocity and the shear rate were effectively limited by the buffer regions and barriers (see the Supporting Information, Fig. S4, for the design).

**Figure 5:**
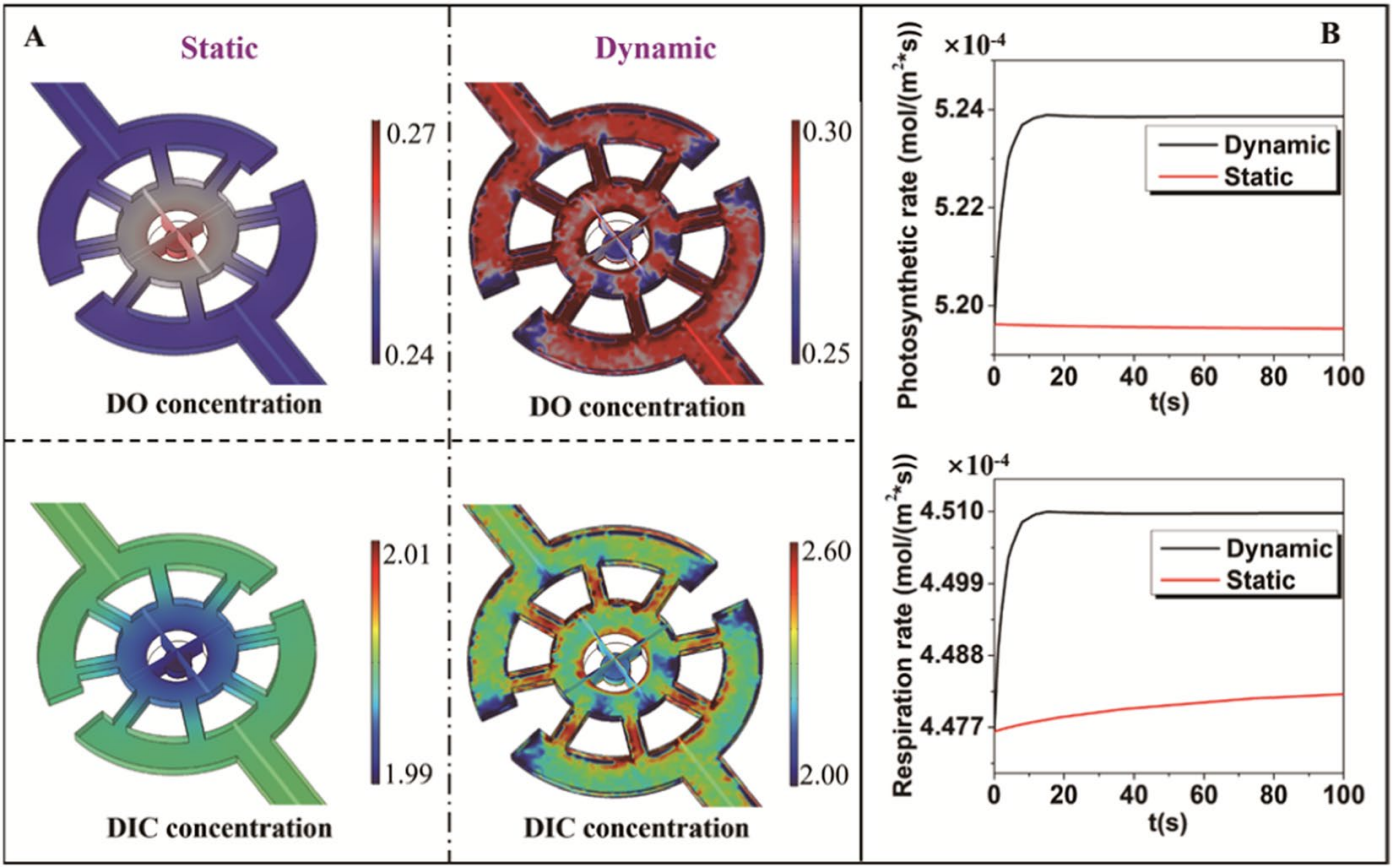
(**A**) The DO and DIC distributions under the dynamic and static conditions. Right columns under the dynamic condition with flowing on and left columns under the static condition with flowing off. (**B**) The photosynthetic and respiration rates were displayed as a function of the culture time under the dynamic and static conditions.

Regarding the metabolic process of the coral polyps, DO and DIC play important roles in the respiration and photosynthesis^35^. The lack of DO and DIC can limit the growth of the corals with the symbiotic algae during the culture^16^. Therefore, a suitable microenvironment in the 3D microchip must be presented for the respiration and photosynthesis of the coral polyps. The respiration and photosynthesis of the coral polyp were analyzed based on the mass transfer and the dynamical model of the metabolism, as shown in Eq. (1.4), (1.5) and (1.6) in the Supporting Information, respectively. And the detailed parameters used in the model were summarized in the Supporting Information (Table S1). Figure 5A showed the simulated results of the concentration distributions of DO and DIC by solving the laminar flow and mass transfer coupled equations. In order to highlight the difference between the microfluidic chip and the traditional method, the static and dynamic conditions were analyzed. The faster metabolite consumption and production were found under the static condition than those under the dynamic condition, which then led to the accumulation of DO and the reduction of DIC for the coral polyp in the central area of the microwell. The DIC concentration was lower than the initial value, 2 ${\rm{mol}}\,{{\rm{m}}}^{-3}$, under the static condition, and became even lower and lower as the culture went on over longer simulation time. This ultimately led to the separation of zooxanthellae from the coral polyps (see the Supporting Information, Fig. S1). However, the situation was different under the dynamic condition, where the steady gradient distributions for DO and DIC were obtained because the liquid flow promoted the substance exchange in our microchip. The DO and DIC concentrations at the center area were about 0.26 and 2.2 ${\rm{mol}}\,{{\rm{m}}}^{-3}$, respectively. This implied that the flow environment could enhance the mass transfer process, leading to the on-demand environment for the coral polyp culture. Furthermore, the respiration and photosynthetic rates over time were evaluated under the dynamic and static conditions, as shown in Fig. 5B. It should be noted, although the simulation period was short, the trends over longer time can clearly be predicted in our simulation result. The simulation indicated that the respiration and photosynthetic processes can quickly reach steady states under the dynamic condition and keep at higher levels. For the static condition, the larger scale simulation results are shown in Supporting Information (Fig. S6). Under the static condition, for the photosynthetic process (red curve, top panel in Fig. 5B), the photosynthetic rate decreased gradually over time. This explained why the long-term culture of the coral polyps can’t be performed *in vitro* under the static condition. where the energy supply from the photosynthetic process constantly decreased for even prolonged time and the coral polyps had to die eventually^16^.

From the above simulation results, it could be concluded that our microchip could implement the balance between the high DO and high DIC mass transfer and render the low fluid shear rate. The results of steady respiration and photosynthesis rates suggested that the microchip platform could present an open interface to study the biological behaviors for the coral polyps in response to additional environmental stimuli.

## Discussion

**Figure 6:**
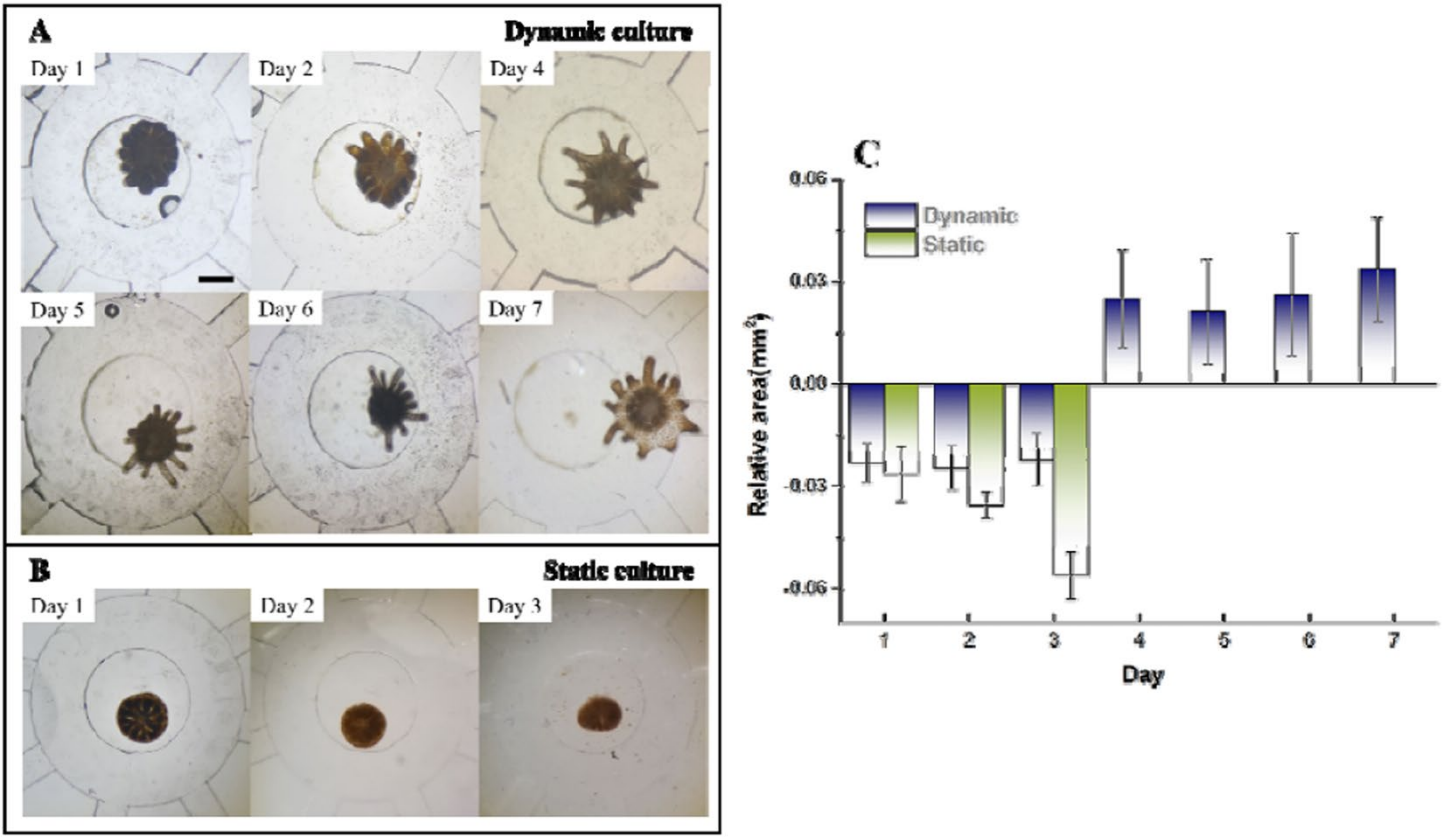
Optical microscopic images of *P. damicornis* polyps cultured under the dynamic (**A**) and static (**B**) conditions, and the relative sizes over time (**C**). Under the dynamic condition, FASW kept flowing in the microchip at a flow rate of 30 ${\rm{\mu }}{\rm{L}}\,{{\rm{\min }}}^{-1}$, whereas under the static condition, FASW was stagnant. Room temperature was 26 °C with a 12/12 h illumination/dark cycle. The relative area was calculated by referring to the initial size. The scale bar is 1 mm.

As is known, a coral polyp is the basic clonal biological unit of reef corals. Current investigations on coral polyps from colonial corals are limited by their poor viability *in vitro*. The most significant problem here is that individual coral polyps can only survive for a short period of time after the detachment from the coral skeleton. A previous study reported that a coral polyp was induced to detach from the colonial hard coral, which survived for a week^16^. However, using our microfluidic platform, *P. damicornis* polyps could grow and develop with good tissue integrity for more than 15 days. As shown in Figs. 6A and S7, the initial polyp explanted to the microfluidic system was still in a stressful state with frizzy tentacles. The tentacles of the coral polyp rarely relaxed as normal and the boundaries of the tentacles were ambiguous. A large number of zooxanthellae gathered in or even flowed out of the mouth. However, with the one-day recovery at a flow rate of 30 uL min^−1^, the single coral polyp in the microchip could move freely and their tentacles were fully relaxed (Movie S2). In the next few days, the coral polyp grew bigger with better vitality than before (Fig. 6C).

In comparison, under the static condition, the body of the coral polyp began to shrink and extended its mesenteric filaments the next day after the explantation. On the third day, coral cells and zooxanthellae flowed out of the mouth of the coral polyp and eventually caused the death of the coral polyp (Fig. 6B). The possible reason was that the static condition created a diffusion barrier in the microenvironment, limited the DIC supply for the photosynthesis of zooxanthellae and the DO supply for the respiration of the coral polyp, thus resulting in the death of the coral polyp^36^. In addition, the accumulation of metabolites worsened the quality of the surrounding microenvironment, which could also lead to the death of the coral polyp. However, under the dynamic condition, metabolites were continuously removed with the FASW. DO in the FASW can support the respiration, which in turn sustained the photosynthesis, thus creating a balanced microenvironment. This dynamically balanced mode in the microchip facilitated the survival and development of the coral polyp for a longer period of time.

In the long run, we can assume that the microchip system to culture the single coral polyps can be used for the long-term physiological and ecological studies such as the ocean acidification and El Nino effects on the reef corals at a microscopic level. Upon bailing-out, a single mother colony can generate many coral polyps. It will be more convenient and controllable to conduct the experimental study for many cultured coral polyps at the same time in order to retrieve the reliable results in comparison to using small colonies or nubbins as the experimental units^37–39^. For example, it has been reported that the deterioration of the coral colonies under the low pH condition was initiated from the single coral polyps^28^. With the coral bleaching occurring more and more frequently^2^, the studies using single coral polyps as experimental units can be a useful and alternative approach to understand the inherent mechanism. In consequence, such investigations can be carried out with the aid of the coral-polyp-on-chip.

In summary, we established a convenient and assembled microfluidic chip platform to provide a dynamic and controllable microenvironment for the single coral polyp culture. The design based on the microwell and the physical barriers in the chip enhanced the mass transfer efficiency, reduced the impact of the shear force and avoided the accumulation of metabolites. In reward, the single coral polyp survived and developed with good viability over a long period of time, i.e. fifteen days, in comparison to the case under the static culture condition. It was observed that the individual polyp of *P. damicornis* stretched out tentacles *in vitro* when cultured in our microfluidic platform, similar to the cultured case in the glass aquarium where the coral colonies developed healthy^31^. Moreover, because this coral polyp-on-chip is a modular device which can be standardized, it can easily be combined with various techniques, such as vibrational spectroscopy, high performance liquid chromatography-mass spectrometry (HPLC-MS), nuclear magnetic resonance (NMR), and the super resolution microscopy for the purpose of studying the coral polyps from different perspectives. Current studies are under way to decipher the biological cues for the single coral polyps under the environmental stressors, for example temperature change and/or ocean acidification.

## Supplementary information

Supporting information.

Move S1.

Move S2.
