# Supplementary material for: A polyp-on-chip for coral long-term culture
Source: Sci Rep. 2020 Apr 24;10:6964. doi: 10.1038/s41598-020-63829-4 (PMC7181858; doi:10.1038/s41598-020-63829-4)
Supplement: Supplementary file 1 — Supporting information. [file 41598_2020_63829_MOESM1_ESM.docx]

Supporting Information

A polyp-on-chip for coral long-term culture

*Ai-Ping Pang, Yongsheng Luo, Chunpeng He*, Zuhong Lu*and Xiaolin Lu**

State Key Laboratory of Bioelectronics, School of Biological Science & Medical Engineering, Southeast University, Nanjing, 210096, Jiangsu Province, P. R. China

The characteristic parameters for DO and DIC transport in the microfluidic chip

The inlet flow rate for the micro-injection pump (*Q*) was 90 μL min^-1^. The Reynolds number to characterize the flow is given by:

$Re=\frac{\rho QL}{\mu S}=0.75$ (1.1)

where $\rho$ is the fluid density (1000$\mathrm{kg}m^{-3}$), *S* is the cross-sectional area of the flow channel ($0.6 \mathrm{mm}^{2}$), $\mu$ is the fluid viscosity (1$mPa s$), *L* is the characteristic dimension of the channel (300 μm). When the Reynolds number is small enough, just like the one in our model (0.75), the velocity distribution in the steady state can be obtained by solving the three-dimensional laminar Navier-Stokes equations.

$\rho\left( u\cdot\nabla\right)\cdot u=\nabla\cdot\left[ -pI+\mu\left( \nabla u+\left( \nabla u \right)^{T} \right) \right]-\rho g$ (1.2)

$\rho\nabla\cdot\left( u \right)=0$ (1.3)

here *u* is the velocity of the fluid (m s^-1^), *p* is the local pressure (Pa) and *I* is the identity matrix.

The DO, DIC transport and consumption for the single coral polyp cultured in the chip can be described by the mass-balance equation.

$\frac{{\partial c}_{i}}{\partial t}+\nabla\cdot\left( -D_{i}\nabla c_{i}+uc_{i} \right)=R_{i}$ (1.4)

here *u* is the fluid velocity, $c_{i}$ is the concentration of the biochemical substances, $R_{i}$ is the reaction rate of the biochemical substances for the coral polyp. To simulate the biological process in the chip, the coral polyp growing in the microwell was regarded as a small reactor with considering the respiration and photosynthesis as two major metabolic processes. The photosynthesis of zooxanthellae can be taken as a function of the light intensity and the concentration of the reactants (biochemical substances) in the fluid. The gross photosynthesis rate should abide by the following equation ^1-3^.

$R_{p}=P_{max}tanh(\frac{E}{E_{k}})\frac{c_{DIC}}{K_{P}+c_{DIC}}$ (1.5)

where $P_{max}$ is the maximum value of photosynthesis, $c_{DIC}$ is the concentration of DIC, $K_{P}$ is the half value of the photosynthesis rate, $E, E_{k}$ *E* and *E_k_* are the photon flux density and a constant related to the photon irradiance, respectively. Besides, the respiration rate (*Rr*) can be considered as the Michaelis-Menten type ^1,4^.

$R_{r}=R_{max}\frac{c_{O_{2}}}{K_{O}+c_{O_{2}}}$ (1.6)

where $R_{max}$ is the maximum respiratory rate, and $K_{O}$ is the half-saturation constant.

Table S1 Parameters for the simulation model.

| Parameters | Values |
| --- | --- |
| Initial DO concentration | (0.25 mmol L^-1^)^4^ |
| Initial DIC concentration | (2 mmol L^-1^)^5^ |
| Single coral polyp maximum photosynthetic rate $P_{\max}$ | (0.73 nmol cm^-2^ s^-1^)^6^ |
| Single coral polyp maximum oxygen consumption rate $R_{\max}$ | (0.53 nmol cm^-2^ s^-1^)^6^ |
| Half-saturation constants of photosynthesis $K_{P}$ | (408 umol L^-1^)^7^ |
| Half-saturation constants of respiration $K_{O}$ | (46 umol L^-1^)^4^ |
| The photon flux density | (350 umol m^-2^ s^-1^)^6^ |
| The photon irradiance constant | (273 umol m^-2^ s^-1^)^8^ |

Coral polyp bail-out with 80% and 20% DMEM concentrations


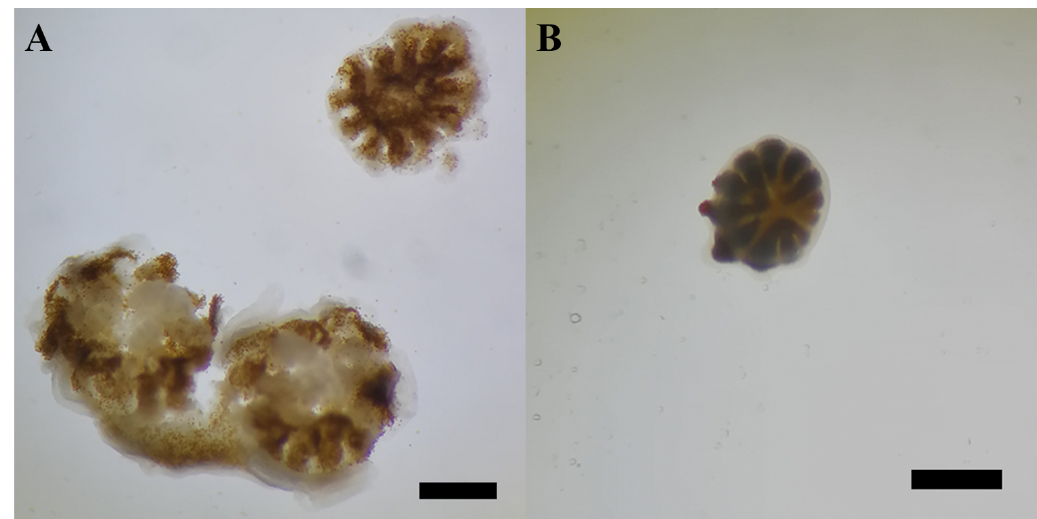


Fig. S1 Initial morphologies of the coral polyps bailed out in 80% (A) and 20% (B) culture mediums. Scale bars: 1$mm$.

Morphologies of single *P. damicornis polyp*


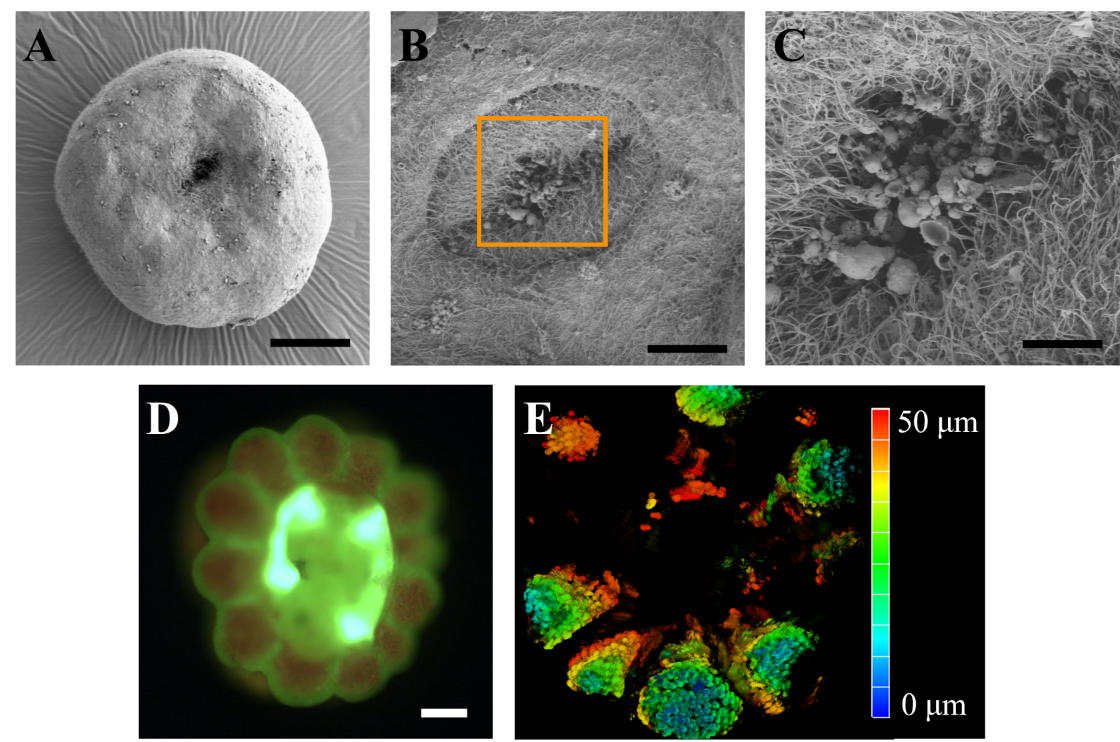


Fig. S2 Images of *P. damicornis* polyp under SEM and LSCM. (A, B, C) SEM images of an intact polyp (A) for the aboral end, (B) polyp’s mouth, and (C) high magnification view of the area marked with the orange rectangle in B . (D) Polyps imaged in CCD mode under LSCM. (E) Depth distribution for overall autofluorescence from both coral native GFP and algal chlorophyll. Scale bars:100 $\mu m$.

**The optimization process of the microfluidic design**

**

**

Fig. S3 The shear rate and Peckert number of the microfluidic chip in terms of the inlet flow rate.

Experimental setup for culturing a single coral polyp


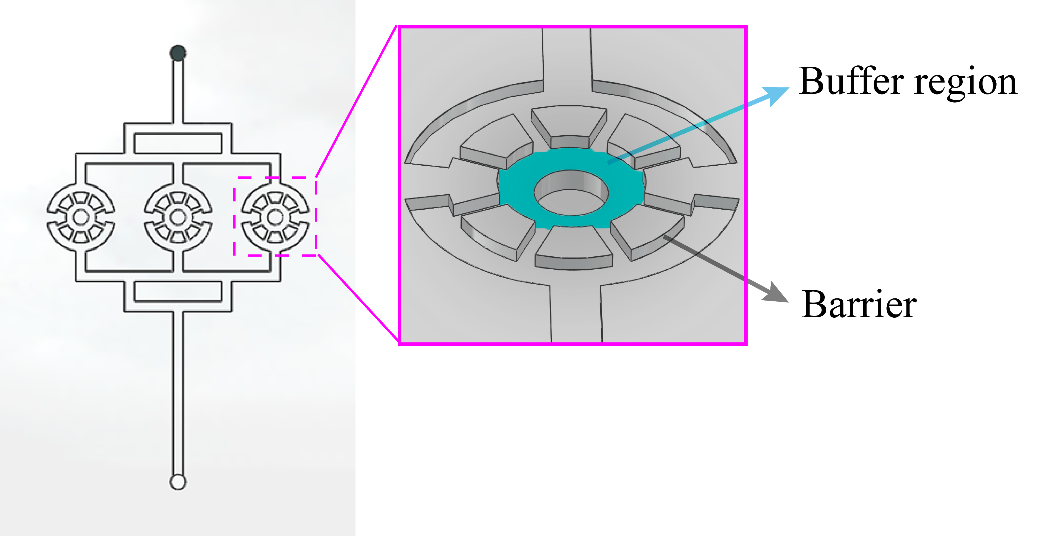


Fig. S4 Schematic shows the experimental setup of the microwell with the barrier to provide the buffer region, thus avoiding the impact of the strong shear force on the coral polyp.


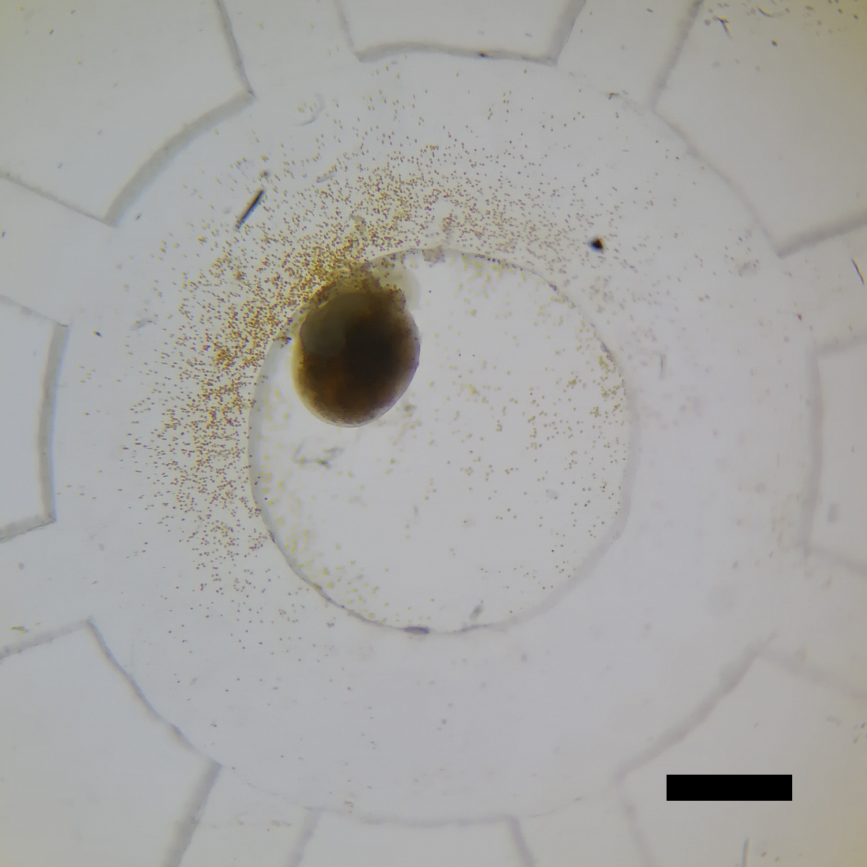


Fig. S5 Optical microscopic image of *P. damicornis* polyp cultured for 72 h under the static condition. Scale bar:1 $\mathrm{mm}$.


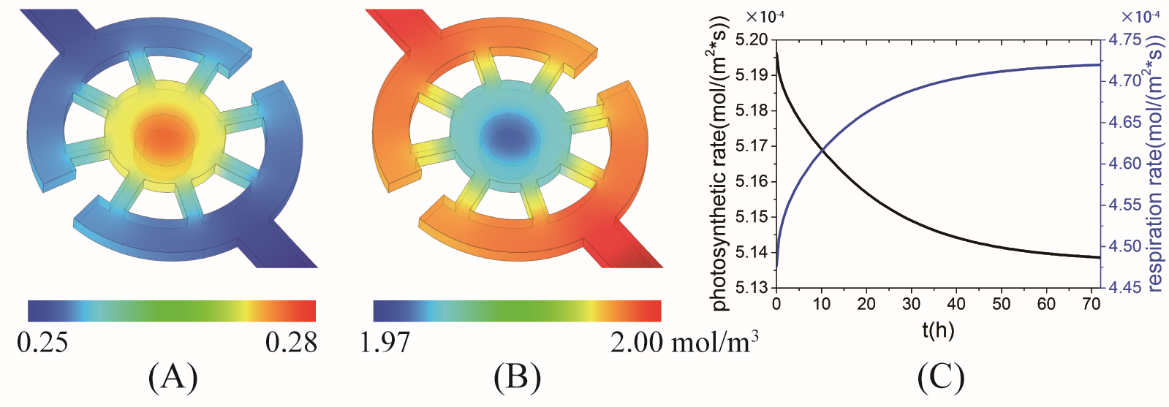


Fig. S6 (A) and (B) are the DO and DIC distributions simulated for two hours under the static condition, respectively. (C) The photosynthetic and respiration rates as a function of time under the static condition (up to three days).


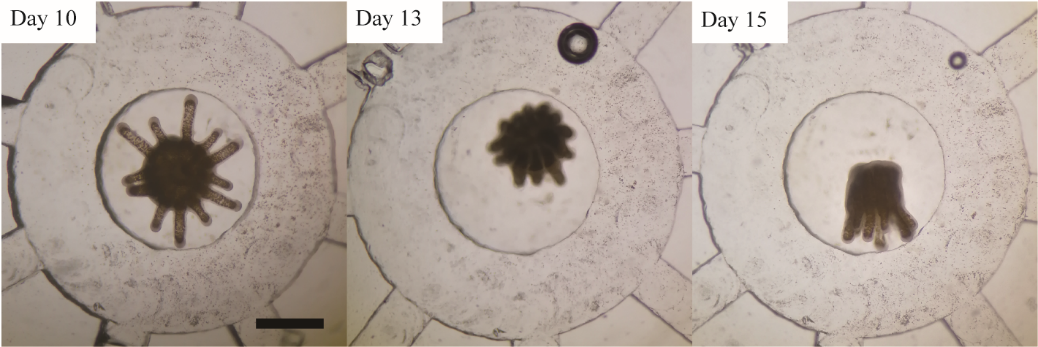


Fig. S7 Optical microscopic images of the coral polyp cultured on days of 10, 13 and 15 under the dynamic condition. The scale bar is 1 mm.

**References**

1 Nakamura T., Nadaoka K. & Watanabe A. A coral polyp model of photosynthesis, respiration and calcification incorporating a transcellular ion transport mechanism. *Coral Reefs* **32**, 779-794, (2013).

2 Burris J. E., Porter, J. W. & Laing, W. A. Effects of carbon dioxide concentration on coral photosynthesis. *Marine Biology* **75**, 113-116 (1983).

3 Aizawa, K. & Miyachi, S. Carbonic anhydrase and CO_2_ concentrating mechanisms in microalgae and cyanobacteria. *FEMS Microbiology Reviews* **39**, 215-233 (1986).

4 Newton, P. A. & Atkinson, M. J. Kinetics of dark oxygen uptake of Pocillopora damicornis. Pacific Science **45**, 270-275 (1991).

5 Aizawa, K. & Miyachi, S. Carbonic anhydrase and CO_2_ concentrating mechanisms in microalgae and cyanobacteria. *Fems Microbiology Letters* **39**, 215-233 (1986).

6 Nakamura, Nadaoka & Watanabe. A coral polyp model of photosynthesis, respiration and calcification;incorporating a transcellular ion transport mechanism. *Coral Reefs* **32**, 779-794 (2013).

7 Goiran, C., Almoghrabi, S., Allemand, D. & Jaubert, J. Inorganic carbon uptake for photosynthesis by the symbiotic coral/dinoflagellate association I. Photosynthetic performances of symbionts and dependence on sea water bicarbonate. *Journal of Experimental Marine Biology & Ecology* **199**, 207-225 (1996).

8 Kühl, M., Cohen, Y., Dalsgaard, T., Jørgensen, B. B., & Revsbech, N. P. Microenvironment and photosynthesis of zooxanthellae in scleractinian corals studied with microsensors for O_2,_ pH and light. Marine Ecology-Progress Series, **117**, 159-172, (1995).
